# An Adaptation of Pavlovian-to-Instrumental Transfer (PIT) Methodology to Examine the Energizing Effects of Reward-Predicting Cues on Behavior in Young Adults

**DOI:** 10.3389/fpsyg.2020.00195

**Published:** 2020-02-14

**Authors:** Raquel Quimas Molina da Costa, Emi Furukawa, Sebastian Hoefle, Jorge Moll, Gail Tripp, Paulo Mattos

**Affiliations:** ^1^D’Or Institute for Research and Education (IDOR), Rio de Janeiro, Brazil; ^2^Neurology Department, Universidade de São Paulo, São Paulo, Brazil; ^3^Human Developmental Neurobiology Unit, Okinawa Institute of Science and Technology Graduate University, Okinawa, Japan; ^4^Institute of Psychiatry, Federal University of Rio de Janeiro, Rio de Janeiro, Brazil

**Keywords:** Pavlovian conditioning, reward anticipation, conditioned stimuli, Pavlovian-to-instrumental transfer, fMRI- functional magnetic resonance imaging

## Abstract

There is growing recognition that much of human behavior is governed by the presence of classically conditioned cues. The Pavlovian-to-Instrumental Transfer (PIT) paradigm offers a way to measure the effects of classically conditioned stimuli on behavior. In the current study, a novel behavioral task, an adaptation of the PIT framework, was developed for use in conjunction with an fMRI classical conditioning task. Twenty-four healthy young adults completed (1) instrumental training, (2) Pavlovian conditioning, and (3) a Transfer test. During instrumental training, participants learned to apply force to a handgrip to win money from slot machines pictured on a computer screen. During Pavlovian conditioning, slot machines appeared with one of two abstract symbols (cues), one symbol was predictive of monetary reward. During the Transfer test, participants again applied force to a handgrip to win money. This time, the slot machines were presented with the Pavlovian cues, but with the outcomes hidden. The results indicated increased effort on the instrumental task, i.e. higher response frequency and greater force, in the presence of the reward-predicting cue. Our findings add to the growing number of studies demonstrating PIT effects in humans. This new paradigm is effective in measuring the effects of a conditioned stimulus on behavioral activation.

## Introduction

Pavlovian, or classical, conditioning refers to an associative process through which previously neutral stimuli acquire motivational significance after repeated pairing with a rewarding or aversive experience (Corbit and Balleine, 2003). When a previously neutral stimulus is repeatedly paired with reward, the stimulus comes to elicit physiological responses and induce appetitive behaviors that are normally associated with reward (Cartoni et al., 2016). Such appetitive classical conditioning is thought to underlie much of everyday human behavior (Bray et al., 2008; Blechert et al., 2016; Cartoni et al., 2016), helping maintain both adaptive and maladaptive behaviors, for example addiction (Hogarth, 2012; Garbusow et al., 2016). A growing number of human-subjects studies have demonstrated the effects of a conditioned cue on attentional bias (Thewissen et al., 2007; Le Pelley et al., 2015, 2016; Leong et al., 2017), choice (Astur et al., 2015; Vogel et al., 2018; Genauck et al., 2019), response speed (Freeman et al., 2014; Asci et al., 2019) and response frequency (Garbusow et al., 2016, 2019; Alarcón and Bonardi, 2019). However, the evidence of behavioral invigoration in the presence of conditioned stimuli in humans is less robust compared to a long history in animal experiments.

In animal experiments, the effects of appetitive classical conditioning are studied using primary rewards, such as food, which elicit innate, reflexive responses (Corbit and Balleine, 2011). Repeatedly providing primary rewards in experiments with human subjects can be challenging and deprivation prior to an experiment (e.g. hunger or thirst) may be required to ensure reward saliency (Freeman et al., 2015; Manglani et al., 2017; Schad et al., 2019). Well-established secondary rewards (such as images of food, money or points) are often used in the place of primary rewards, with the consumption of rewards typically delayed (e.g. accumulating points during, but receiving actual rewards after an experiment). Such procedural constraints can interfere with observation of simple behavioral activation by classically conditioned cues, e.g. approach behavior to the food hopper in animal experiments.

The Pavlovian-to-Instrumental Transfer (PIT) paradigm offers a way to measure the effects of conditioned stimuli on behavior in humans. In a PIT task, the motivational influence of reward-predicting cues is measured through their effects on independently learned instrumental behavior (Cartoni et al., 2016). A PIT task has three components. During Pavlovian conditioning, subjects are exposed to previously neutral cues (e.g. sounds), at least one of which is repeatedly followed by rewards (e.g. food). In a separate instrumental conditioning phase, subjects are trained to engage in behavior (e.g. lever press) to obtain rewards. During the transfer phase, Pavlovian cues are presented while subjects have the opportunity to engage in the previously learned instrumental behavior. If the cues have acquired motivational value, their presence should invigorate the behavioral response (e.g. increased lever pressing). The transfer phase is carried out under extinction conditions, so that instrumental behavior is not modified by ongoing rewards (Levey and Martin, 1991; Corbit and Balleine, 2011). The order of Pavlovian conditioning and instrumental training varies, and similar transfer effects have been observed with either order (Cartoni et al., 2016).

Researchers have used a single excitatory cue (reward-predicting cue) to examine the activating effects of the cue on behavior (Hall et al., 2001; Holland and Gallagher, 2003; Talmi et al., 2008). More complex paradigms have been developed to assess outcome-specific vs. more general behavioral activation effects. Outcome-specific effects are examined by using two excitatory cues, each predictive of a unique reward during Pavlovian conditioning, each reward associated with a unique action during instrumental training (e.g. Corbit et al., 2001; Corbit and Balleine, 2005; Morris et al., 2015). General activation is measured through the effects of a third excitatory cue, predictive of a further unique reward during Pavlovian conditioning, but not associated with an action during instrumental training. The activating effects observed in experiments using a single cue, as described above, have been likened to general (rather than outcome-specific) activating effects, on the basis of lesion studies (Murschall and Hauber, 2006; Corbit and Balleine, 2011).

A number of human PIT studies has been reported in recent years, reflecting growing interest in understanding the effects of classically conditioned cues on behavior. A little over half of these studies have examined appetitive conditioning, with many focusing on addictive behaviors, in relation to food (Pool et al., 2015; Quail et al., 2017a; Seabrooke et al., 2017; van Steenbergen et al., 2017), nicotine (Hogarth, 2012; Hogarth et al., 2015; Manglani et al., 2017) and alcohol (Martinovic et al., 2014; Garbusow et al., 2016; Hardy et al., 2017; Sommer et al., 2017). Some studies used targets of addiction (e.g. smells, pictures, taste) as cues or rewards, and others abstract cues and unrelated rewards (e.g. points, money), to study how the behavioral effects of conditioning vary. Other studies have used PIT paradigms to evaluate how stress and depressed mood affect motivation (Huys et al., 2016; Quail et al., 2017a), or to study the neural correlates of the transfer effects in non-disordered populations (Paredes-Olay et al., 2002; Talmi et al., 2008; Allman et al., 2010; Geurts et al., 2013; Hebart and Gläscher, 2015; Sebold et al., 2016).

In many of these human appetitive PIT studies, the transfer effect is examined through the influence of conditioned stimuli on goal-oriented behavior, most often response frequency or go/no-go response accuracy (Garbusow et al., 2014; Sebold et al., 2016). Outcome-specific transfer effects are most frequently documented (Manglani et al., 2017; van Steenbergen et al., 2017). General behavioral invigoration of classically conditioned cues appears more difficult to demonstrate in humans. Among appetitive PIT studies examining the differential effects of outcome-specific vs. general behavioral activation, we identified only six reporting general transfer effects (Prevost et al., 2012; Watson et al., 2014; Hebart and Gläscher, 2015; Quail et al., 2017b; Alarcón and Bonardi, 2019; Garofalo et al., 2019). A small number of other human PIT studies used designs with a single excitatory cue, and reported transfer effects (Talmi et al., 2008; Lovibond and Colagiuri, 2013; Colagiuri and Lovibond, 2015; Pool et al., 2015).

Efforts to understand the effects of appetitively conditioned stimuli in humans extend to fMRI studies. A small number of studies have used simple classical conditioning paradigms to study the neural correlates of reward-predicting cues (e.g. Furukawa et al., 2014; Klucken et al., 2016). In these studies, as no behavioral response was required, conditioning effects on behavior were not measured. In other studies, participants were instructed to make a behavioral response following a reward-predicting cue, with fast accurate responding a measure of subjects’ motivation (e.g. Knutson et al., 2000; O’Doherty et al., 2007). In these latter paradigms, it is difficult to determine if observed BOLD responses reflect anticipation of acting to obtain reward or anticipation of the reward itself.

We developed a novel behavioral task, an adaptation of the PIT framework with a single excitatory cue, for use in conjunction with fMRI classical conditioning tasks (Furukawa et al., 2020). This new paradigm facilitates the measurement of BOLD responses in anticipation of the reward itself together with recording of the behavioral effects of classical conditioning. Instrumental behavior was established prior to fMRI scanning; participants learned to grip a hand dynamometer to earn monetary rewards. Pavlovian conditioning took place in the MRI scanner. A previously neutral cue was paired with monetary reward outcome, another cue with no-reward outcome. No behavioral response was required between the cue and outcome presentation, allowing observation of BOLD signals to reward anticipation, unconfounded by anticipation of acting to obtain rewards. Transfer effects, handgrip responses in the presence of the now reward-predicting and non-reward-predicting cues, were evaluated following the scanning session. This provided a behavioral measure of classical conditioning effects, important in interpreting functional brain activations to reward predicting cues, that is neural evidence of classical conditioning effects.

A number of factors influenced task design. Instrumental responding required behavioral invigoration, i.e. effort. Motivated behavior has been characterized as *effortful* action to obtain desirable outcomes (Chong et al., 2016; Bortolini et al., 2017). Thus, grip strength and grip frequency were both used as measures of instrumental responding. Pavlovian/classical conditioning was optimized for fMRI scanning. Only two cue types were used; one excitatory cue associated with probabilistic reward and a second cue associated with non-reward. This ensured sufficient trials of each cue type during fMRI scanning, while keeping the total time in the scanner tolerable. This methodology does not allow for a distinction between general vs. specific transfer effects, however, this was not the purpose of the current study. Our goal was the measurement of behavioral invigoration in the presence of a reward-predicting cue. A simulated gambling task was utilized to provide an ecologically valid justification for the use of probabilistic reward and maintain participants attention, especially in the MRI scanner.

In this paper, we describe the new task in some detail and present data on the behavioral effects of the reward-predicting cue. Based on the available human PIT studies using a single excitatory cue, we expected to observe behavioral activation in the presence of the reward-predicting cue following the Pavlovian conditioning phase. Behavioral activation would be reflected in greater force applied to the hand dynamometer and increased frequency of grips, in the presence of the reward- versus non-reward-predicting cue during the transfer phase.

## Materials and Methods

Ethical approval for the study was obtained from the ethics committee of the D’Or Institute for Research and Education (IDOR), Brazil. All participants were volunteers and provided written consent.

### Participants

The study included data from 24 typically developing young adults (Mean age = 27.50 SD = 3.75 Mean Estimated IQ = 104.63 SD = 5.97; Mean years of education = 17.00 SD = 1.77; 54.16% female), recruited at local universities and through IDOR researchers’ personal contacts. All participants belonged to middle and upper socioeconomic classes^1^. Participants completed a demographic and background questionnaire, an abbreviated IQ test [Wechsler Abbreviated Scale of Intelligence Vocabulary and Block Design subtests (Weschler, 1999; Trentini et al., 2014)] and structured interviews with a psychiatrist [Kiddie-Schedule for Affective Disorder and Schizophrenia-PL (Brasil, 2003) and Structured Clinical Interview (Del-Ben et al., 2001)]. The inclusion criteria for the study were: no major depressive or bipolar disorder, attention-deficit hyperactivity disorder, neurological disorder, current drug use or psychotic symptoms. This excluded individuals with conditions associated with altered motivational processes, i.e. depressed or elevated mood, hyperactivity and impulsivity symptoms, addictive behavior (Tripp and Wickens, 2008; Whitton et al., 2015; Conzelmann et al., 2016).

### Experimental Task

The task included Instrumental training, Pavlovian conditioning and a PIT test. The task was programmed using Presentation^®^ version 16.5, by Neurobehavioral Systems Inc. Participants completed the Pavlovian conditioning inside a 3T Achieva scanner (Philips Medical Systems, Netherlands), with task stimuli shown using an LCD display and mirror adapted to the head coil. The instrumental training and transfer test were conducted outside the MRI scanner with the stimuli presented on a computer screen. Instrumental responses were measured using a hand dynamometer^2^ calibrated with a BIOPAC MP160 System with AcqKnowledge software (Biopac^®^ Systems Inc). Participants were told they would receive their winnings (monetary rewards) accumulated throughout the experiment in the form of a gift card at the end of the experiment.

Before instrumental training, each participant’s maximum grip force was measured. Participants were asked to grip the dynamometer as hard as possible three times, from these grips their mean maximum force was calculated. Participants then practiced gripping the hand dynamometer to spin a “slot machine” pictured on a computer screen, as many times as they wanted, for 10 s.

**FIGURE 1:**
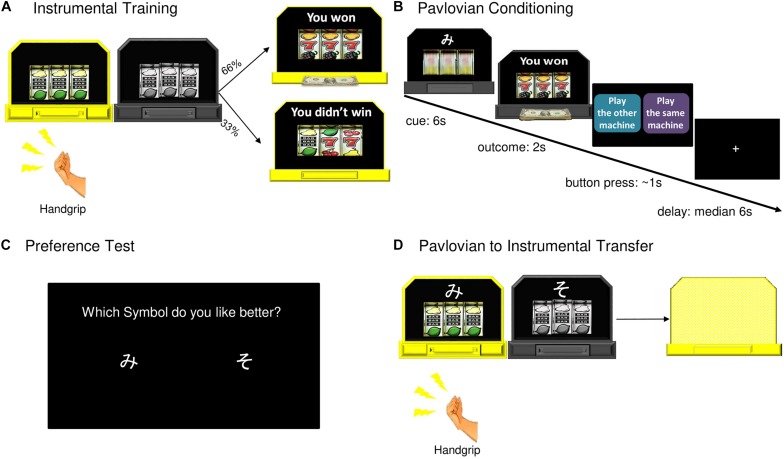
Pavlovian-to-Instrumental Transfer task. **(A)** During instrumental training, one machine became available (lit up) for a participant to play (grip the hand dynamometer). Twelve ten second blocks with machines alternating, a 66% chance of winning on either machine. **(B)** During Pavlovian conditioning, one of two abstract symbols was paired with each slot machine. Machine A yielded reward 66% of the time (CS+, 44 trials), Machine B was never associated with winning (CS–, 44 trials). **(C)** After Pavlovian conditioning, participants were asked which of the two symbols (cues) they preferred. **(D)** During the transfer test, CS+ and CS– were displayed with slot machines, the outcomes “hidden” from the participants. There were six ten second blocks for each machine.

Next, participants were told they would see two slot machines pictured side by side on a computer screen (Figure 1). At the beginning of instrumental training the two slot machines were both gray. One machine at a time lit up and became available for a participant to play for 10 s before switching off. The two machines were identical during this phase of the study. The participants were told they could grip the dynamometer as many times as they wanted while a machine was lit and the more times they played the more chances they had to win. Each machine was programed to start spinning when the grip force reached 40 or 60% of the participant’s mean maximum force, requiring them to exert effort. The force required alternated to prevent participants from modulating their grip to a set level (Talmi et al., 2008). There was a 66% chance of winning on either machine. There were 12 ten second blocks with 5 s rest periods between each block. If participants failed to apply the necessary force to spin the slot for three consecutive seconds, the block ended. Machine availability alternated, with all participants playing each machine for six blocks. The instrumental training phase lasted for approximately 3 min.

During Pavlovian conditioning (in the scanner), participants were told to watch the computer play the slot machines, and that the earnings would be added to their gift card. One of two abstract symbols (Cue A or Cue B; two Japanese hiragana letters) was displayed on top of each slot machine. Machine A (with Cue A) yielded reward 66% of the time (CS+). Machine B (with Cue B) was never associated with winning (CS−). The slot machine spun for 6 s, and an outcome was displayed for 2 s. After each trial, participants were asked to “suggest” to the computer whether to play the same machine (with the same cue) or the alternative machine by pressing one of two buttons placed in their right hand, within a 1 s response window. Participants were told that the computer may or may not follow their suggestion. This “choice” was designed to maintain participants’ attention during conditioning and did not influence machine presentation. Actual presentation order was semi-random with the constraint that there be no more than four consecutive trials with presentation of the same machine/cue, this was the same for all participants. Pavlovian conditioning lasted for approximately 27 min and included 44 trials of Cue A (CS+) with reward outcome, 22 trials of Cue A with non-reward outcome, and 44 trials of Cue B (CS−). At the end of this phase, participants were asked which of the two symbols (cues) they preferred.

The transfer test was identical to instrumental training with the following exceptions: (a) the reward and non-reward cues from the Pavlovian conditioning phase were displayed with their associated slot machines and (b) the outcomes were “hidden” from the participants to allow examination of the behavioral effects of the cues in the absence of ongoing contingencies. Participants were told the computer would record their earnings, which would be added to their gift card (see Supplementary Figure S1 for the instructions). There were 12 ten second blocks for all participants: six blocks with Machine A (CS+) and six blocks with Machine B (CS−), becoming available in a semi-random order, with the constraint of no more than two consecutive trials with the same machine. At the end of the transfer test, the total money earned during the experiment was displayed for the participants on the screen (total amount = R$ 0.33 × the number of wins). All participants received gift cards in the total amount earned, which ranged from R$30 to R$50.

### Data Analysis

To obtain a baseline measure of the participants’ instrumental learning, the frequency of grips above the maximum force threshold and the force applied (including the grips below the threshold) were recorded for each of the two slot machines. During the transfer test, the frequency of grips and force levels were again recorded. Repeated measures GLM was used to compare within-subject effects of the two machines (CS+ vs. CS−) and blocks (six blocks per machine). Participants’ responses during the Pavlovian phase were also examined to assess their preference toward the CS + (log10[percent stay after CS + /(1 – percent stay after CS+)]) versus CS− (log10[percent stay after CS−/(1 – percent stay after CS−)]). The percentage of missed response windows following all three trial types, i.e. CS+ followed by reward, CS+ followed by non-reward, and CS− followed by non-reward, were also compared.

## Results

### Instrumental Training Phase

**FIGURE 2:**
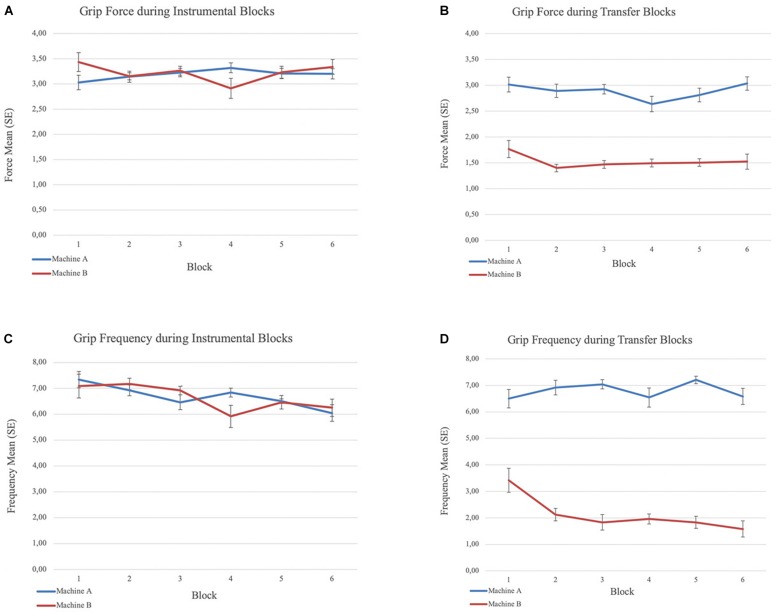
Grip force and frequency means and standard errors (within-subject) during the instrumental and transfer phases. **(A)** Mean grip force across blocks during instrumental phase. **(B)** Mean grip force across blocks during transfer phase. **(C)** Mean grip frequency across blocks during instrumental phase and **(D)** Mean grip frequency across blocks during transfer phase.

All participants learned to apply force to the handgrip to spin the slot machine during instrumental training. We did not expect that participants’ behavior would be different across the two slot machines. Repeated measures ANOVA confirmed there was no significant difference in the mean force level applied, or the frequency of grips, between the two machines. The frequency of grips per block declined over the six blocks on both machines (*F*(2.33,65.40) = 3.28, *p* < 0.05, Greenhouse-Geisser correction for the repeated-measures block effect), likely reflecting fatigue. Grip force level remained stable suggesting continued engagement in the task throughout instrumental training (Figure 2).

### Pavlovian Conditioning Phase

A paired sample *t*-test on the logit-transformed values showed a greater proportion of suggested stays following the CS + (88%) versus the CS− (14%) trials, *t*(23) = 8.25, *p* < 0.001. Moreover, the participants were significantly more likely to suggest staying on the same machine following CS+ non-reward trials (80%) than CS− non-reward trials (14%), *t*(23) = 5.72, *p* < 0.001. This would seem to indicate that participants’ suggestions were made based on the cues presented.

All participants responded, i.e. suggested which machine to play next, during the 1 s window, more than 75% of the time, indicating adequate attention to task. Participants were less likely to miss the response window when the CS+ was followed by reward outcome (7%) than non-reward outcome (54%), *t*(23) = −15.32, *p* < 0.001. They were also less likely to miss responding after CS− trials (all non-reward outcome) (8%), than after CS+ non-reward trials (54%), *t*(23) = −14.54, *p* < 0.001.

All participants indicated that they preferred Cue A (CS+) over Cue B (CS−) when questioned after completing Pavlovian conditioning.

### Pavlovian-to-Instrumental Transfer Phase

Repeated measures ANOVA yielded a main effect of machine (CS) for force, *F*(1,115) = 25.36, *p* < 0.001. Participants applied greater force on Machine A (displayed with CS+) than Machine B (displayed with CS−) across blocks (Figure 2). Repeated measures ANOVA also yielded a main effect of machine (CS) for the frequency of above-threshold grips *F*(1,115) = 54.76, *p* < 0.001, and a block × machine interaction effect, *F*(3.87,89.05) = 10.65, *p* < 0.001, Greenhouse-Geisser correction for the repeated-measures block effect^3^. Participants responded more frequently on Machine A (CS+) than Machine B (CS−). The frequency of grips was maintained over the 6 blocks on Machine A (CS+) but declined on Machine B (CS−). Further, participants gripped the dynamometer with the required level of force at least once (frequency of grips above the maximum force threshold ≥1) during each block 97.9% of the time in presence of the CS+, but only 38.9% of the time in the presence of the CS− (*x*^2^(1, *N* = 288) = 116.07, *p* < 0.001).

## Discussion

The current study evaluated the motivating effects of reward-predicting cues on human behavior. A new task was developed, using a PIT framework. The task included three phases; instrumental training, Pavlovian (classical) conditioning, and a transfer test. Pavlovian conditioning trials were optimized for fMRI. In this initial study to assess the task’s efficacy, the behavioral effects of appetitive conditioning were observed. The presence of the reward-predicting cue motivated participants to engage in effortful behavior. The results show the task is useful for evaluating the effects of classical conditioning on behavioral activation in humans, when conditioning takes place in situations where movement restrictions are required or preferred and approach behavior during conditioning is difficult to observe.

In the current task, during instrumental training, participants learned to apply force to a hand dynamometer to spin slot machines to earn monetary rewards. Response frequency was observed to decline over blocks. Participants acquired instrumental behavior quickly and appeared to be motivated to engage in the behavior. The instrumental phase lasted long enough for participants to experience some fatigue. During Pavlovian conditioning, participants demonstrated a behavioral preference for the slot machine associated with the reward-predicting cue, i.e. were more likely to suggest playing this machine again. Following conditioning, all participants selected the symbol coupled with reward when asked for their preference. During the transfer test, in the presence of the reward-predicting cue, participants applied more force to and gripped the hand dynamometer more frequently, compared to the non-reward cue. The number of grips, of the required force, was stable across response blocks in the presence of the reward-predicting cue. Grip frequency declined over non-reward cue blocks.

This task was developed to facilitate completion of Pavlovian conditioning in an MRI scanner. The Pavlovian conditioning phase allows observation of BOLD responses to a reward-predicting cue and reward delivery. Many other fMRI studies examining BOLD effects associated with reward anticipation use a paradigm that requires behavioral responses following a reward-predicting cue. The current task allowed us to use a simple classical conditioning task and measure BOLD responses to reward-predicting cues unconfounded by anticipation to act to obtain rewards. A single reward cue was used, rather than multiple cues associated with different outcomes or magnitudes of wins and losses, to avoid complex outcome valuations or odds calculations. This also allowed sufficient presentations of each cue-outcome pair for image acquisition.

Using a single reward cue did not allow for outcome-specific vs. general transfer to be examined separately during the transfer phase. However, differentiating these effects was not a goal in developing this task. Animal studies suggest the excitatory effects of a single cue relate to general invigoration rather than to specific goal-directed behavior (Cartoni et al., 2016). In the current task, instrumental responding required participants to apply force. This response modality was selected to quantify participants’ physiological effort. Motivation, as expressed in behavior, has been characterized as engaging in effortful action to obtain desirable outcomes (Chong et al., 2016; Bortolini et al., 2017). Compared to other response modalities, such as speeded repeated responses on keys or buttons, the force requirement provides clearer evidence of effortful responding/behavioral invigoration.

The transfer effects in the current study were evaluated as the difference in behavioral activation in the presence of the CS+ vs. CS−, rather than as a change from an active baseline with a neutral cue introduced during the transfer phase. The aim of the study was to examine the behavioral effects of the reward cue and non-reward cue shown during the Pavlovian conditioning. In the current study, the observed transfer effects might be due to CS+ energizing and/or CS− inhibiting effects on behavior (Quail et al., 2017a). A neutral baseline would have helped clarify this. The force applied and the frequency of above-threshold grips in the presence of CS+ during the transfer phase were not greater than during the instrumental phase. This is likely due to ceiling effects as participants were observed to grip hard during the instrumental phase. Force requirements to spin the slots machines were based on participant’s maximum grip strength, spinning only when at least 40 or 60% of the mean force was applied. These procedural requirements likely led participants to apply significant force during the instrumental training phase. In previous PIT studies, greater transfer effects were reported when rates of responding during the instrumental phase were lower (Cartoni et al., 2016). In the current study, the maintenance of effortful behavior in the presence of the CS+, even when participants were likely fatigued, argues for its energizing effects.

Slot machines were used throughout all three phases of the task. This provided an ecologically valid justification for the use of probabilistic reward and continuity across the phases. The task made sense to the participants without requiring extensive instructions or explanations. However, this may have made it easier for participants to narratively link the reinforcement contingencies of the two machines during Pavlovian conditioning to the transfer phase, i.e. that cues signal the differential availability of rewards for instrumental behavior. The behavioral effects observed during the transfer phase could represent observational learning or the reward values of the machines being updated during the Pavlovian conditioning phase. All participants explicitly indicated a preference for the CS+ over the CS− following Pavlovian conditioning. Whether transfer effects would have been observed without such cognitive awareness is not known. Some studies have demonstrated awareness of the reinforcement contingencies affects transfer effects; successful or stronger effects have been reported for participants who explicitly identify the contingencies (Lovibond and Shanks, 2002; Talmi et al., 2008; Jeffs and Duka, 2017). In human PIT studies, it is likely that transfer effects include some degree of explicit behavioral activation, informed by the conditioned cue, in this study the decision whether to grip forcefully. Force level measurement in the current task included even slight application of pressure to the handgrip, potentially capturing some degree of implicit behavioral activation, although teasing apart the degree of implicit vs. explicit activation is not possible. When the conditioning/training length or session number is limited, as in many human studies, it may be difficult to establish strong transfer effects of classical conditioning without some explicit awareness of the cues and their meaning.

The current findings are consistent with a growing number of recent studies demonstrating PIT effects in humans (Seabrooke et al., 2017; Sommer et al., 2017; Verhoeven et al., 2018; Vogel et al., 2018) and provide additional evidence for behavioral invigoration in the presence of a classically conditioned stimulus. The simplicity of the task allows for its use in fMRI studies, and possibly with a range of participants, including clinical populations, who may demonstrate limited tolerance for spending time in an MRI scanner (Corbit and Balleine, 2005; Martinovic et al., 2014; van Steenbergen et al., 2017). However, the task has some limitations as discussed above. Additionally, behavioral responses required during the Pavlovian conditioning phase (machine suggestion) may have created an expectation for the following trials. This could affect BOLD responses during the Pavlovian phase and behavioral conditioning effects. Symbols associated with CS+ and CS− were not counter-balanced across and within participants for consistency in the stimuli presented in the MRI. The possibility of a pre-existing preference for the symbol associated with CS+ cannot be excluded. The response frequency and force were similar between two machines during the instrumental trials, providing no evidence of a pre-existing place preference. The utility of the current paradigm needs to be further examined through its use in multiple fMRI studies, which would document whether the classical conditioning task reliably results in differential BOLD responses to reward cues vs. non-reward cues and reward outcomes vs. non-reward outcomes.

In developing a human experimental task based on animal studies, unique procedural constraints should be considered, with design specifications optimized for study aims. Continued efforts are required to establish and improve tasks for measuring basic but powerful general motivating effects of classically conditioned cues in humans. Altered sensitivity to reward-predicting cues has been hypothesized to contribute to a range of psychiatric conditions. Better understanding of the neural and behavioral effects of Pavlovian cues in humans may improve understanding of the neurobiology of these disorders and help refine treatment strategies.

## Data Availability Statement

The datasets generated for this study are available on request to the corresponding author.

## Ethics Statement

The studies involving human participants were reviewed and approved by the Ethics Committee of the D’Or Institute for Research and Education (IDOR), Brazil. The participants provided their written informed consent to participate in this study.

## Author Contributions

All authors contributed to the conception and design of the study, responsible for writing the manuscript, read, and approved the submitted version. RC, SH, and PM collected data. RC and EF performed statistical analysis.

## Conflict of Interest

PM was on the speakers’ bureau, received travel awards and/or acted as consultant for Shire/Takeda in the last 5 years. The remaining authors declare that the research was conducted in the absence of any commercial or financial relationships that could be construed as a potential conflict of interest.

## Footnotes

1 http://www.abep.org/

2 www.biopac.com

3 Due to significant variability in the data, i.e. some participants applied very little force during blocks with Machine B (CS−), with no grips exceeding thresholds during some blocks, the fixed effects were checked in the mixed-model analyses adjusted for repeated effects (unstructured covariance). Significant main effects of machine (CS) type were observed for grip force (*F*(1,24) = 26.46, *p* < 0.001) and frequency (*F*(1,24) = 57.14, *p* < 0.001). The block^∗^machine interaction effect was also observed for grip frequency (*F*(5,24) = 4.34, *p* < 0.01).
