# Supplementary material for: An Adaptation of Pavlovian-to-Instrumental Transfer (PIT) Methodology to Examine the Energizing Effects of Reward-Predicting Cues on Behavior in Young Adults
Source: Front Psychol. 2020 Feb 14;11:195. doi: 10.3389/fpsyg.2020.00195 (PMC7034436; doi:10.3389/fpsyg.2020.00195)

Supplementary Figure 1. Instructions (English translation) presented at the beginning of the transfer phase.

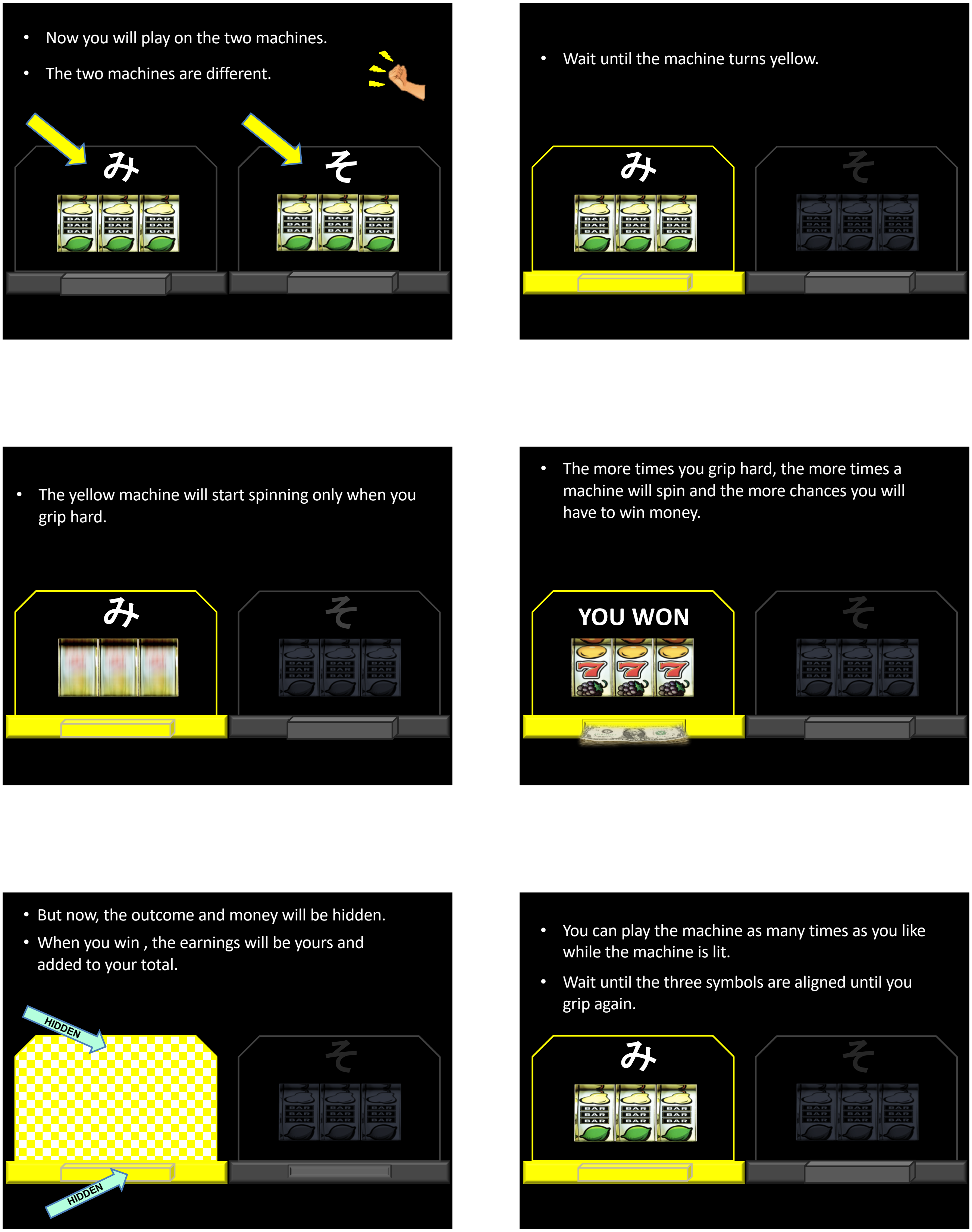

- There will be time for you to rest between trials.

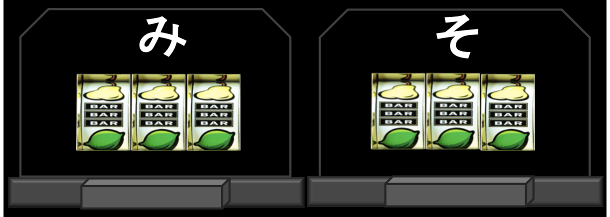

- If you don’t grip hard, the machine will turn off.
- But the machine will be turn on (become yellow) again after a while.
- Let’s try and see what happens when you do not grip hard. When the machine turns yellow, don’t grip hard.

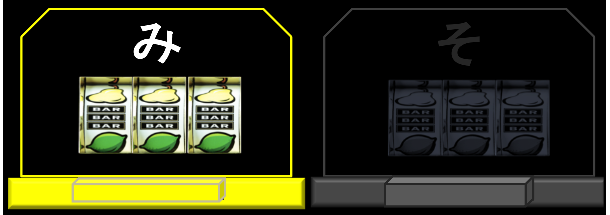

SIMULATION

- Sometimes you win, sometimes you don’t.

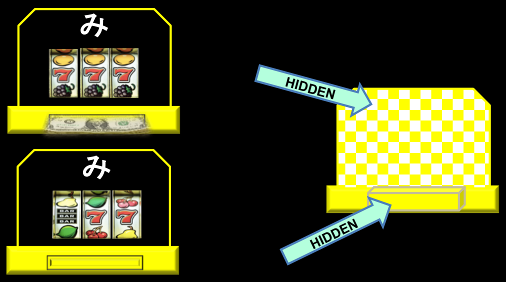

- The outcome and money will be hidden.

- You will have 3 minutes to play the game.
- Maximize your earning!
- Let’s begin.

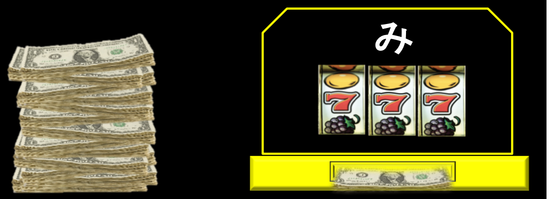

Supplement: Supplementary file 1 [file Data_Sheet_1.PDF]
